# A True Estimate

**Published:** 1858-12

**Authors:** 


### A TRUE ESTIMATE

Of HALL'S JOURNAL OF HEALTH may be arrived at as follows: There is, perhaps, not a single intelligent newspaper reader in the United States with whom the name of our periodical is not, more or less, familiar, in consequence of the great frequency with which it is quoted. But, as often as our journal is quoted, by the great partiality of our exchanges, a very large number of our articles (published three, and four, and five years ago) are going the round of the papers, without any paternity being given. We have often read an anonymous article nearly through, and concluded the author must have been a very intelligent person indeed, until we came to some word or phrase which reminded us that it was our own, when we were satisfied that our conclusions were—correct.

Our Subscribers, as a means of finding out whether we have told all we know, or that there is a plenty of the same sort left, are respectfully invited to take the journal another year.

